# Reciprocal Crosstalk Between YAP1/Hippo Pathway and the p53 Family Proteins: Mechanisms and Outcomes in Cancer

**DOI:** 10.3389/fcell.2019.00159

**Published:** 2019-08-09

**Authors:** Nitin Raj, Rakesh Bam

**Affiliations:** ^1^Department of Radiation Oncology, Stanford University School of Medicine, Stanford, CA, United States; ^2^Department of Radiology, Stanford University School of Medicine, Stanford, CA, United States

**Keywords:** Hippo pathway, YAP, P53, P63, ΔNp63, P73, apoptosis, cancer

## Abstract

The YAP1/Hippo and p53 pathways are critical protectors of genome integrity in response to DNA damage. Together, these pathways secure cellular adaptation and maintain overall tissue integrity through transcriptional re-programing downstream of various environmental and biological cues generated during normal tissue growth, cell proliferation, and apoptosis. Genetic perturbations in YAP1/Hippo and p53 pathways are known to contribute to the cells’ ability to turn rogue and initiate tumorigenesis. The Hippo and p53 pathways cooperate on many levels and are closely coordinated through multiple molecular components of their signaling pathways. Several functional and physical interactions have been reported to occur between YAP1/Hippo pathway components and the three p53 family members, p53, p63, and p73. Primarily, functional status of p53 family proteins dictates the subcellular localization, protein stability and transcriptional activity of the core component of the Hippo pathway, Yes-associated protein 1 (YAP1). In this review, we dissect the critical points of crosstalk between the YAP1/Hippo pathway components, with a focus on YAP1, and the p53 tumor suppressor protein family. For each p53 family member, we discuss the biological implications of their interaction with Hippo pathway components in determining cell fate under the conditions of tissue homeostasis and cancer pathogenesis.

## Introduction

The Yes-associated protein 1 (YAP1; henceforth referred to as YAP), the main effector of the Hippo signaling pathway, has been reported to regulate multiple physiological processes such as tissue regeneration, morphogenesis, metabolism and tumorigenesis as well as a variety of cellular processes spanning cell-cell communication, cell cycle regulation, signal transduction and cytoskeletal remodeling (Zhao et al., 2010; Hansen et al., 2015; Meng et al., 2016; Panciera et al., 2017; Ardestani et al., 2018). YAP activation is predominantly controlled by the components and upstream regulators of Hippo pathway. The core Hippo pathway molecule mammalian Ste20-like kinases 1/2 (MST1/2) phosphorylates Large tumor suppressor kinase 1/2 (LATS1/2), which then phosphorylates YAP, resulting in its interaction with 14-3-3 protein, and sequestration in the cytoplasm for degradation by proteasomes (Yu et al., 2012; Meng et al., 2016). When the Hippo/LATS1/2 pathway is turned off, YAP can shuttle into the nucleus and promote multiple transcriptional programs. YAP is a potent transcriptional co-activator that lacks direct DNA-binding activity but modulates gene expression by binding to other transcription factors. YAP might act either as oncogene or tumor suppressor depending on its binding partner and its subcellular localization (Bertini et al., 2009). YAP can function as an oncogene through its interactions with the TEA domain transcription factors (TEAD). The TEAD family of transcription factors contain an N-terminal DNA-binding domain and a C-terminal region responsible for YAP interaction (Vassilev et al., 2001; Anbanandam et al., 2006). TEAD factors directly bind to YAP and mediate YAP-induced gene expression to modulate YAP target genes involved in cell proliferation, invasion and suppression of apoptosis (Zhao et al., 2008; Holden and Cunningham, 2018). Indeed, YAP is frequently amplified or hyperactivated in a number of human solid tumors (Zanconato et al., 2016). On the other hand, YAP has been reported to function as a tumor suppressor in breast cancer and hematological malignancies by promoting apoptosis in these contexts. In breast cancer, YAP locus is a site of frequent loss of heterozygosity and consequently breast tumors demonstrate significant loss of YAP protein (Yuan et al., 2008). In case of hematological malignancies such as multiple myeloma, leukemia and lymphomas, low YAP levels in the tumor cells prevents ABL1-kinase induced apoptosis in response to DNA damage (Cottini et al., 2014).

The p53 protein plays a pivotal role in tumor suppression, as evidenced by its inactivation in over half of human cancers (Olivier et al., 2010). p53 is a DNA-binding transcription factor that suppresses tumor growth through activation of target genes involved in diverse biological processes (Bieging et al., 2014). p53 is a cellular stress sensor that responds to diverse stress signals, such as DNA damage, hypoxia, and oncogene expression, by inducing cell-cycle arrest, cellular senescence, or apoptosis, as a measure to restrain neoplasia and shape organ development (Vousden and Prives, 2009; Bieging et al., 2014). In response to cellular stress signals, p53 is displaced from its negative regulators, such as MDM2 E3 ligase that promotes p53 ubiquitination and proteasomal degradation, leading to its stabilization and activation. Activated p53 can bind to consensus p53 response elements in the genome and induces the transcription of a plethora of target genes that regulate diverse cellular processes to ultimately suppress tumorigenesis (Marine and Lozano, 2010; Kaiser and Attardi, 2018). Like many eukaryotic transcription factors, p53 has a modular protein domain structure that is critical for its function as a transcription activator and tumor suppressor (Brady et al., 2011). p53 has two N-terminal transactivation domains, a central DNA binding domain and a C-terminal oligomerization domain (Chillemi et al., 2017). A significant majority of mutations in p53 occur in the central DNA-binding domain, which suggests that function of p53 as a transcription factor is crucial for tumor suppression (Olivier et al., 2010). Early diversification of p53 through domain re-organization has allowed evolution of p53 family proteins to have various functional roles through regulation of diverse transcriptional programs in response to environmental and biological cues. Two p53 homologs, p63 and p73, that share remarkable homology in DNA sequence as well as in protein structure also function as transcription factors that bind to specific DNA response elements (Belyi et al., 2010). The p53 family shares many overlapping functions such as induction of apoptosis in response to DNA-damage (Levrero et al., 2000). However, the existence of extensive structural variability within the family determines unique roles for p63 and p73 in the regulation of development and differentiation (Murray-Zmijewski et al., 2006; Botchkarev and Flores, 2014). Together, the p53 gene family is involved in transcriptional regulation of development, differentiation and cell response to stress.

Added complexity of crosstalks between the members of p53-family and Hippo pathway indicate that multiple molecular interfaces of these pathways interact, physically and functionally, to determine cell fate. In response to activation of upstream signaling modules by the ever-changing tissue microenvironment, YAP and p53 can dynamically co-ordinate to generate various gene expression signatures to mount an appropriate cellular response. Several other upstream factors regulate YAP and p53 expression and activity in relation to fine-tuning of stem cell self-renewal, apoptosis, and proliferation, which are often imbalanced in pathological states of cancer, fibrosis, metabolic disorders, and inflammation (Kruse and Gu, 2009; Yu and Guan, 2013). For instance, upstream signaling proteins, such as G-protein coupled receptors, receptor tyrosine kinases, wingless/integrated (Wnt) protein, tight junction proteins, and extracellular matrix factors influence the roles of not only the Hippo pathway components but some of them also have consequences on p53 function (Dotto, 2009; Ghosh et al., 2011; Lappano and Maggiolini, 2011; Pickup et al., 2014; Yoh and Prywes, 2015; Nowell and Radtke, 2017; Totaro et al., 2018). In this review, we will shed light on the major intersections where members of p53 and Hippo pathways assemble, affecting YAP protein stability, nuclear localization and transcriptional activity. Conversely, the effect of Hippo signaling components, primarily YAP, in apoptotic and oncogenic functions of p53 family members will be outlined based on the evidence from current literature. How these important physical and genetic crosstalks can modulate the physiological roles of p53 family members and YAP in different cell and disease contexts will also be discussed.

### *TP53* Status Serves as the Molecular Switch for YAP Function as Tumor Suppressor or Oncogene

Many components of the YAP/Hippo and p53 pathways functionally and physically interact to govern cell-fate decisions. In this section, we will examine the published literature reporting the major points of crosstalk between the YAP/Hippo and p53 pathways and discuss the biological outcomes associated with each interaction. We will first discuss experimental evidence demonstrating cooperativity, in tumor suppression, between YAP/Hippo and wild-type p53 under conditions of cellular stress. In the subsequent subsection, we will discuss how aberrant p53 enables the YAP/Hippo pathway to promote oncogenesis.

### Wild-Type p53 and YAP/Hippo Pathway Cooperate in Cellular Stress Responses and Homeostasis

The Hippo pathway and wild-type p53 cooperate, at many levels, as tumor suppressors to induce senescence and apoptosis in response to stress conditions. YAP can directly bind to *TP53* gene promoter and upregulate p53 expression leading to apoptosis during hepatocellular carcinoma chemotherapy. In turn, p53 can bind to the *YAP* promoter and upregulate YAP expression, establishing a positive feedback loop. Thus, YAP and p53 support each other to modulate chemosensitivity in hepatocellular carcinomas (Bai et al., 2013). The YAP/Hippo pathway can impinge upon p53 function, through LATS1/2’s ability to bind and inhibit MDM2, the major inhibitor of p53, leading to p53 stabilization, as well as through the regulation of modulators of p53-mediated apoptosis (Visser and Yang, 2010; Furth and Aylon, 2017). This crosstalk is triggered by several genomic stresses, such as oncogene expression, cytokinesis failure, tetraploidy and replication stress, and can lead to p53-dependent cell cycle arrest and apoptosis as discussed below.

**FIGURE 1:**
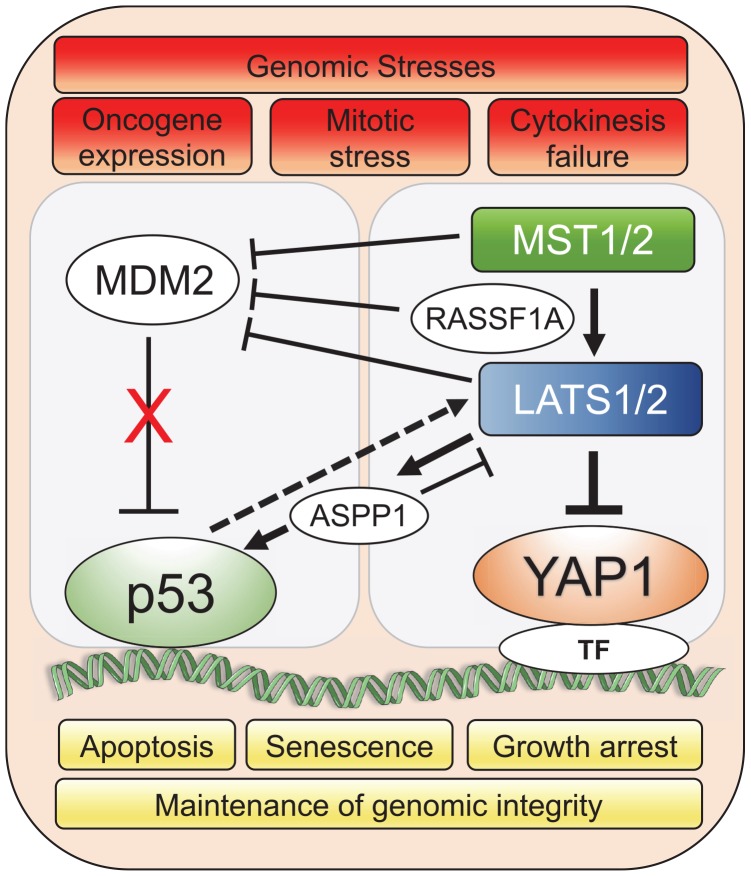
YAP1/Hippo and wild-type p53 pathways coordinately maintain genomic stability in response to stress. Genomic stresses such as oncogene expression, mitotic stress and cytokinesis failure trigger a crosstalk between Hippo and p53 pathways leading to mainly p53-dependent cellular growth arrest, senescence or apoptotic transcriptional programs to preserve genomic integrity. Upstream Hippo pathway components, MST1/2 and LATS1/2, modulate p53 activity through co-operation with intermediary factors such as ASPP1 (Aylon et al., 2010; Vigneron et al., 2010) and RASSF1A (Song et al., 2008) to inhibit MDM2 E3 ligase that targets p53 for proteasomal degradation. Solid lines represent direct interaction and dotted line represents transcriptional upregulation. TF represents the diverse array of transcription factors such as TEAD that the Hippo pathway effector, YAP1, associates with for spatio-temporal control of cell fate under genomic stress.

LATS1/2 bind and inhibit MDM2 which results in wild-type p53 activation and prevents accumulation of polyploid cells during mitotic stress. Specifically, LATS2 activates p53 in the nucleus and transcription of *LATS2* is positively regulated by p53, leading to p53-dependent cell cycle arrest and apoptosis (Aylon et al., 2006, 2010). In response to oncogenic H-Ras expression in human lung embryonic fibroblasts, LATS2 expression and nuclear translocation is upregulated, resulting in its inhibitory binding to MDM2, p53 stabilization and concomitant induction of apoptosis and senescence (Aylon et al., 2009). In the event of cytokinesis failure, cells with genome doubling and instability also undergo apoptosis through stabilization of p53 by Hippo pathway. LATS2 stabilizes p53 protein through direct inhibition of MDM2 while concomitantly inactivating YAP to resist the propagation of tetraploid cells (Ganem et al., 2014). In response to genotoxic stress, Ras association domain-containing protein 1 (RASSF1A) activates MST2 and LATS1, negatively regulating MDM2 and preventing p53 degradation (Guo et al., 2007; Song et al., 2008). Additionally, RASSF1A selectively induces the YAP target gene *ANKRD1*, an epigenetically silenced tumor suppressor gene in human tumors, which promotes p53 growth suppressive programs by destabilization of MDM2 (Jimenez et al., 2017). The apoptotic activity of p53 can also be indirectly governed by LATS2 through regulation of the apoptosis-stimulating protein of p53-1 (ASPP1). Specifically, in the context of oncogenic stress, LATS2 can phosphorylate ASPP1 and drive its nuclear localization, where it shunts p53 to the proapoptotic gene promoters (Aylon et al., 2010). Interestingly, ASPP1 can also inhibit LATS1 mediated phosphorylation of YAP in the cytoplasm, which allows increased YAP translocation to the nucleus and cell proliferation (Vigneron et al., 2010). Overall, the LATS-MDM2-p53 axis serves as a novel cell cycle checkpoint that is critical for the maintenance of proper chromosome number and genome integrity (Figure 1).

Loss of function of critical components of either the Hippo or p53 pathway can shape organ development as well as have unique repercussions in stress response leading to disease. Inactivation of LATS1/2 kinase leads to a transient YAP-mediated hypertranscription and proliferation of neural progenitor cells during brain development, which triggers replication stress and DNA damage, setting-off p53-mediated apoptosis (Lavado et al., 2018). LATS1/2-deletion-induced hyper-activation of YAP also triggers p53-dependent senescence and death in primary hepatocytes during liver development (Lee et al., 2016). However, loss of YAP activity can sensitize cells to apoptosis that can be p53-dependent or independent. YAP silencing in MCF-7 breast cancer cell line stabilizes wild-type p53 in response to cisplatin treatment, while this silencing in p53 null SAOS2 osteosarcoma cells promotes cell cycle progression but increases sensitivity to DNA damage by cisplatin (Ferraiuolo et al., 2018). Together, these studies indicate that the reciprocal regulation of YAP/Hippo and p53 pathways and their cooperativity in cellular responses to genomic stresses are observed in both development and disease.

### Perturbation of p53 Function Enables YAP/Hippo Pathway Driven Oncogenesis

Alterations of the *TP53* tumor-suppressor gene is one of the most frequent events in tumorigenesis (Olivier et al., 2010). Either loss-of-function (via missense mutation or locus deletion) or gain-of-function mutations in the *TP53* coding sequence are associated with cancer initiation and progression (Muller and Vousden, 2013). A significant body of literature has established that an aberrant p53 pathway potentiates YAP mediated tumorigenesis. In lung cancer, loss of p53 in mutant KRAS^G12D^ expressing cells leads to increased YAP nuclear localization and activity, suggesting that KRAS^G12D^ induced tumorigenesis is promoted by YAP in cells that have lost p53 tumor suppressor activity (Mao et al., 2017). Suppression of KRAS in *Kras^Lox–STOP–Lox–G12D^;p53^flox/flox^* murine lung cancer model promoted cell survival through YAP activity suggesting that YAP plays a compensatory role upon loss of KRAS and p53 signaling (Shao et al., 2014). Interestingly, YAP can also overcome the KRAS addiction of p53-deficient pancreatic ductal adenocarcinoma (PDAC). PDAC is the most common malignancy of the pancreas and is the fourth leading cause of cancer-related deaths worldwide with a 5-year survival rate of only 8% (Siegel et al., 2016). A study by Kapoor et al. (2014), showed that YAP amplification-driven overexpression enables KRAS^G12D^-independent tumor relapse and maintenance in mouse and human PDAC. Moreover, inactivating mutations of p53 and loss of tumor suppression compounded by the lack of LATS1/2 activity leads to tolerance of genomic instability and tetraploidy in telomerase-immortalized retinal epithelial cells through uncontrolled YAP activity (Vittoria et al., 2018). These observations highlight tight regulation of YAP signaling in the presence of wild-type p53 to prevent oncogenic cell transformation, while contextual loss of p53 function results in uncontrolled YAP oncogenic activities.

**FIGURE 2:**
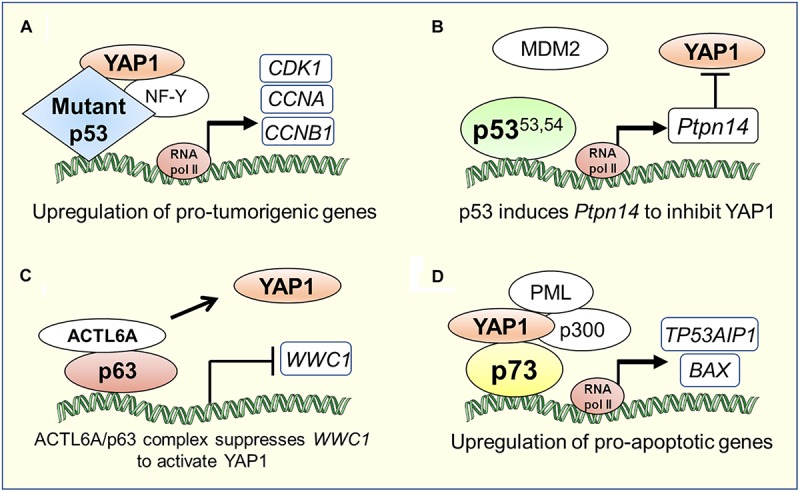
Transcriptional co-ordination between YAP1/Hippo and p53 family proteins in tumorigenesis or apoptosis. **(A)** YAP1 can directly bind to mutant p53 and induce a pro-tumorigenic transcriptional program (Di Agostino et al., 2016). **(B)** Super-tumor suppressor p53^53.54^, a stable p53 mutant due to its diminished ability to interact with MDM2, hyperactivates *Ptpn14* expression that negatively regulates YAP1 in pancreatic cancer (Mello et al., 2017). **(C)** p63 physically interacts with the chromatin-remodeling factor, ACTL6A, to suppress the expression of *WWC1*, a negative regulator of YAP1 nuclear translocation, which leads to tumorigenesis in head and neck cancer (Saladi et al., 2017). **(D)** YAP1 recruits factors such as PML and p300 to the p73 pro-apoptotic target gene promoters (Strano et al., 2005; Lapi et al., 2008).

“Gain-of-function” mutations of p53 are widely known to promote cancer pathogenesis (Shetzer et al., 2016). Recent studies have uncovered another pro-oncogenic mechanism of action of YAP in cancers harboring mutations in the *TP53* gene. A study by Di Agostino et al. (2016), showed that YAP protein physically interacts with human tumor derived mutant p53 (carrying R175H, R273H, R280K or C194D mutations; henceforth collectively referred to as mtp53), inducing the expression of several pro-oncogenic genes and potentiating mtp53’s pro-proliferative transcriptional activity. Using Gene Set Enrichment Analysis to search for associations between genes regulated by mtp53 and collection of gene signatures denoting activation of transcription factors and signaling pathways in breast cancer cell lines, it was discovered that mtp53 and YAP share a common transcriptional program. Furthermore, chromatin immunoprecipitation assays revealed that mtp53 and YAP were bound to NF-Y target oncogenes, *CCNA*, *CCNB1* and *CDK1* promoter sequences (Figure 2A). Depletion of mtp53 or YAP down-regulated the expression of these genes and markedly slowed the growth of breast cancer cells (Di Agostino et al., 2016). Interestingly, in some instances, aberrant Hippo signaling can lead wild-type p53 to acquire the functional capability of oncogenic mtp53. Loss of LATS1/2 in breast cancer leads to conformational changes in wild-type p53 and to a reduced phosphorylation of p53 at Ser15 and Ser315, driving wild-type p53 to a mtp53-like state. This altered p53 conformation affects the p53 protein interactome and promotes cell migration through upregulation of PTGS2 in breast cancer cells along with increased YAP activity in the absence of LATS1/2 (Furth et al., 2015).

The oncogenic activities of mtp53 are also potentiated by intermediary factors that influence Hippo signaling. In glioblastoma and breast cancer cells, mtp53 enhances PI3K/AKT2-mediated phosphorylation of WASP-interacting protein (WIP), an actin cytoskeleton-associated protein, that promotes YAP stability and cancer stem cell survival (Escoll et al., 2017). Upon metabolic cues activating mevalonate pathway, SREBP, a transcription factor that regulates cholesterol metabolism, cooperates with mtp53 to trigger YAP transcriptional program and upregulates key metabolic effector genes of p53 for cancer-stem cell self-renewal (Sorrentino et al., 2014). Additionally, in a human gastric cancer cell line, MGC-803, harboring *TP53* mutations, it was shown that knock-down of long intergenic non-coding RNA p21 (lincRNA-p21), a direct transcriptional target of p53, induced epithelial to mesenchymal transition state and metastasis by elevating YAP expression suggesting that lincRNA-p21 represses YAP expression downstream of p53 (Chen et al., 2017). Studies on an interesting hyperactive “super-tumor suppressor” p53 mutant (p53^*W*53*Q,F*^^54^^*S*^; henceforth referred to as p53^53,54^), harboring double point mutations in the critical hydrophobic residues within the second transactivation domain of p53, have helped uncover a tyrosine phosphatase called PTPN14, an inhibitor of YAP, as a central component of the p53 tumor suppression pathway (Michaloglou et al., 2013; Raj and Attardi, 2017). Recent studies by Mello et al. (2017) demonstrated that p53^53,54^ hyperactivates the expression of *Ptpn14*, a p53 transcriptional target, to suppress induction of PDAC. PDAC originates from the pancreatic ductal or acinar cells, which go through myriad of genetic alterations to give rise to a highly invasive disease (Ying et al., 2016). Global genomic characterization of PDAC has identified activating mutations in *KRAS* oncogene as the driver mutation in 95% of cases and inactivating mutations in *TP53* tumor suppressor gene in ∼75% of cases underscoring the importance of these mutations in PDAC development (Jones et al., 2008; Ying et al., 2016). While it is clear that p53 plays a critical role as a barrier to PDAC development, the exact mechanism of how p53 acts in this context is still lacking. Through overexpression and knockdown of PTPN14 in pancreatic cancer cells, Mello et al. (2017) demonstrated that the p53-PTPN14 axis drives PDAC tumor suppression by inhibition of YAP function. First, PTPN14 overexpression promoted YAP cytoplasmic localization and inhibition of proliferation in pancreatic cancer cells. Second, knockdown of PTPN14 enhanced colony growth in PDAC cells and this phenotype was attenuated upon treatment of these cells with verteporfin, a YAP inhibitor, indicating that PTPN14 negatively regulates YAP to induce growth arrest in PDAC cells (Figure 2B). Interestingly, previous studies have shown that PTPN14 not only inhibits YAP by directly binding to it, but also by binding to LATS1, augmenting its kinase activity, and negatively affecting YAP translocation to the nucleus (Wilson et al., 2014). Thus, upstream regulators of both p53 and YAP namely MDM2 and LATS1, respectively, can impinge upon the Hippo pathway output by coordinately modulating cellular function of PTPN14. Overall, these studies revealed the existence of a transcriptionally coordinated crosstalk between p53 and YAP through intermediary factors. In summary, YAP/Hippo and p53 pathways engage in pervasive reciprocal crosstalk that allows for mutual modulation and integration of function between these critical tumor suppressor pathways.

### ΔNp63 Cooperates With YAP to Promote Oncogenesis

Another p53 family member, p63, can deputize the YAP/Hippo signaling in cell fate decisions. p63 can be mainly categorized into two isoforms: the full-length transactivation domain (TA) isoform, TAp63, and the amino-deleted ΔN isoform (Murray-Zmijewski et al., 2006; Su et al., 2013; Chen et al., 2018). The TAp63 isoform structurally resembles full-length p53 and is capable of inducing cell cycle arrest and apoptosis in response to DNA damage (Flores et al., 2002). Conversely, ΔNp63 isoform exerts oncogenic properties by acting in a dominant-negative manner to counteract the transcriptional activities of p53, TAp63, and TAp73 tumor suppressors (Flores, 2007). Very few reports have uncovered connections between the TAp63 and YAP/Hippo pathway. For example, one report showed that the TAp63 isoform can function as tumor suppressor in breast cancer as loss of TAp63 promotes transition of mammary epithelial cells into tumor-initiating cells through expression of mammary stem cell-related gene signature, partly via the upregulation of TAZ, a YAP paralog and transducer of the Hippo pathway (Su et al., 2017). On the contrary, there exists an extensive body of literature demonstrating that ΔNp63 isoform can uniquely influence YAP activity in both developmental settings and tumorigenesis through physical and genetic interactions with YAP as discussed below.

### ΔNp63 Binds YAP to Regulate Cellular Differentiation and Apoptosis in a Variety of Contexts

ΔNp63 is shown to physically interact with YAP and mediate YAP function in maintenance and self-renewal of adult lung basal stem cells, which regulate epithelial size and architecture of normal airways (Zhao et al., 2014). Through interactions with ΔNp63, YAP can act as an important barrier for phenotypic plasticity in lung cancer. YAP-ΔNp63 interaction can block YAP-TEAD mediated transcriptional repression of S100A7, a factor that is important for the transition process of lung adenocarcinoma to squamous carcinoma trans-differentiation in lung cancer cells (Li et al., 2017; Wang et al., 2017). Conversely, YAP represses ΔNp63 via transcriptional regulation of ZEB2 expression, to inhibit squamous cell trans-differentiation in Lkb1-deficient lung cancer cells (Gao et al., 2014).

Physical association between ΔNp63 and YAP has also been reported in keratinocytes as well as in a head-and-neck squamous cell carcinoma, contexts where ΔNp63 and YAP are frequently overexpressed and amplified (Tomlinson et al., 2010). YAP-bound ΔNp63 protein is stabilized due to protection from its E3 ligase, ITCH, mediated degradation (Chatterjee et al., 2010). Strikingly, in both of these contexts, UV irradiation induced the interaction between ΔNp63 and YAP in a JNK-kinase dependent fashion. Interestingly, YAP protects keratinocytes from UV irradiation, while in head-and-neck cancer YAP promotes UV-induced apoptosis (Tomlinson et al., 2010). In contrast, cisplatin treatment in head-and-neck carcinoma induces c-Abl, a tyrosine kinase, that phosphorylates ΔNp63α, resulting in its increased binding to YAP, leading to protection from cisplatin-induced apoptosis (Yuan et al., 2010). These reports indicate that the ΔNp63-YAP interaction could play a dual role in DNA damage-induced apoptosis in a context-dependent manner.

Without its physical association with YAP, ΔNp63 can regulate YAP/Hippo pathway in cooperation with other factors indirectly affecting YAP nuclear translocation in squamous carcinoma. Saladi et al. (2017) discovered negative regulation of WWC1, the inhibitor of YAP nuclear localization, by the physical interaction of p63 with a chromatin remodeling factor- ACTL6A, resulting in activation of YAP signaling, induction of regenerative state, and tumorigenesis in head-and-neck squamous cell carcinoma (Figure 2C). Consistently, ACTL6A and a YAP-regulated transcriptional programs are significant determinants of poor overall survival for head-and-neck carcinoma.

In summary, p63 and YAP/Hippo signaling components cooperate in tumorigenesis, chemotherapy resistance, cell death, and regulation of stem cells in a cell-context dependent manner. In particular, the relation of p63 with YAP is ΔNp63 isoform-dependent and based on the published literature, it can be concluded that the ΔNp63 determines outcome of Hippo pathway activation directly through physical association with YAP or indirectly through upregulation of YAP nuclear translocation.

### The YAP-p73 Complex Potentiates p73-Dependent DNA Damage-Induced Apoptosis

Like p53, in response to a variety of genotoxic stresses, including DNA damage and oncogene activation, p73 is activated and induces cell cycle arrest and apoptosis (Jost et al., 1997; Zaika et al., 2001; Flores et al., 2002; Murray-Zmijewski et al., 2006). Unlike p53, p73 is only rarely mutated in cancer but it is a bona fide tumor suppressor, being able to induce cell cycle arrest and apoptosis partly via direct protein-protein interaction with YAP. In this section we will discuss the literature that has established connections between p73 and YAP in stress induced apoptosis.

p73 induces apoptosis by transcriptionally upregulating the expression of pro-apoptotic target genes such as *BAX, PUMA, NOXA* and *TP53AIP1* (Costanzo et al., 2002; Melino et al., 2004; Soond et al., 2007; Martin et al., 2009). In response to DNA damage, YAP functions as a transcriptional coactivator of p73 and induces p73-mediated apoptosis (Strano et al., 2001, 2005). YAP and p73 interact via the WW domain of YAP and PPPY motif of p73 (Strano et al., 2001). Specifically, the terminal tyrosine residue in the PPPY motif of p73 is required for association with and coactivation by YAP (Strano et al., 2001). YAP potentiates p73-mediated transactivation by modulating both p73 protein stability and post-translational modifications. Under apoptotic conditions, the formation of the YAP-p73 complex stabilizes p73 by inhibiting its proteasome mediated degradation by the E3 ubiquitin ligase, ITCH. Mechanistically, YAP competes with ITCH for binding to p73 via the PPPY domain on p73 (Rossi et al., 2005; Levy et al., 2007). Additionally, YAP stimulates p73 transactivation potential by enhancing p73 protein acetylation, via recruitment of p300 acetyltransferase (Strano et al., 2005). Under apoptosis triggered by anti-cancer drugs, YAP recruits both promyelocytic leukemia (PML) and p300 transcriptional coactivators, concomitantly, at specific p73 apoptotic target genes such as *TP53AIP1* and *BAX*, to promote p73-dependent apoptotic response (Figure 2D) (Strano et al., 2005; Lapi et al., 2008).

The activity of the YAP-p73 complex is regulated by multiple molecular mechanisms including upstream Hippo pathway components. The YAP-p73 interaction is negatively regulated by LATS- and Akt-mediated phosphorylation of YAP at Ser127 (Basu et al., 2003; Oka et al., 2008). Under normal conditions in unstimulated cells, YAP phosphorylation promotes its dissociation from p73, retention in the cytoplasm and degradation by the proteasome (Basu et al., 2003). Specifically, LATS1-induced YAP Ser127 phosphorylation generates a 14-3-3 binding site on YAP, which induces its cytoplasmic retention, thus inhibiting its co-transcriptional activity. Conversely, in response to DNA-damaging agents such as cisplatin and γ-irradiation, c-Abl directly phosphorylates YAP at Tyr357, which stabilizes YAP protein, enhances its affinity to p73 and selectively coactivates p73 pro-apoptotic target genes such as *BAX* and *TP53I3* (Levy et al., 2008; Keshet et al., 2015). Apoptosis inducing Fas death receptor signaling pathway can also influence YAP-p73 complex formation through the actions of the RASSF1A tumor suppressor. RASSF1A associates with MST2/LATS1 kinase complex, stimulates the phosphorylation of YAP allowing its translocation to the nucleus, binding to p73, and induction of transcription of proapoptotic *PUMA* gene (Matallanas et al., 2007). Another interesting mechanism of YAP-p73 complex regulation is via a proapoptotic autoregulatory feedback loop that exists between YAP, p73 and their transcriptional target PML tumor suppressor. Using chromatin immunoprecipitation, it was demonstrated that PML is a direct transcriptional target of YAP-p73 complex. In turn, PML mediates YAP sumoylation and stabilization, enhancing p73 activity and promoting apoptosis (Lapi et al., 2008).

The YAP-p73 complex plays a pivotal role in eliciting apoptosis in several disease contexts. This complex has been reported to activate pro-apoptotic genes in response to DNA damage signaling downstream of cancer therapy drugs such as cisplatin in colorectal cancer, DNA-damage in hematological malignancies, and Fas signaling in breast cancer as well as in neurodegenerative diseases such as Alzheimer’s (Matallanas et al., 2007; Lapi et al., 2008; Zhang et al., 2011; Cottini et al., 2014). Interestingly, p73-mediated cell death is attenuated by a dominant-negative isoform of YAP in transcriptional repression-induced atypical death of cortical neurons in Huntington’s disease and amylotropic lateral sclerosis (Hoshino et al., 2006; Morimoto et al., 2009). Overall, YAP modulates p73-mediated transcription of pro-apoptotic genes in response to DNA-damage signaling.

## Conclusion and Perspectives

Given the frequent perturbation of YAP/Hippo and p53 pathway activity in human cancer, it is unsurprising that these tumor suppressor pathways are coordinated on multiple levels. Loss of this coordination opens the door for tissue overgrowth, tumorigenesis, and many other diseases. The transcriptional regulator YAP, a key effector of the Hippo signaling, is situated at the cross-roads of the Hippo and p53 pathways. Crosstalk between the p53 family members and YAP can either elicit tumor suppressor or oncogenic effects depending on the functional status and available ratios of p53 protein family.

YAP lacks DNA-binding activity and hence it must interact with a DNA-binding transcription factor to regulate target gene expression. When activated, YAP translocates to the nucleus and interacts through its WW domain with the PPxY motifs of diverse transcription factors to drive multiple transcriptional programs. It is this property of YAP that is frequently harnessed by the PPxY motif containing members of the p53 family to elicit growth control via cellular processes such as differentiation, cell cycle regulation, and apoptosis in response to genotoxic stresses. YAP directly interacts with PPxY containing p53 family members, p73α, p73β, and TAp63α, but not with members that lack this motif, p53 wild-type, p63γ, and p73γ. The differential interaction pattern between YAP and p53 family members is likely to be a function of upstream signaling with different post-translational modifications serving to modify YAP structure, localization, and protein stability. Unlike p63 and p73, wild-type p53 regulates YAP function in an indirect manner, through the physical interactions of other components of the two pathways, namely MDM2 E3 ligase, LATS kinase, ASPP1 and PTPN14. Together, the direct interactions of YAP with p63/p73 and the LATS-MDM2-p53 axis help maintain genomic integrity and restrain neoplasia. Certain tumor associated mutant p53 proteins and p63 isoforms (ΔNp63) can hijack YAP transcriptional activity and switch the biological output of the p53-family/YAP interaction from pro-apoptotic activators to pro-tumorigenic and metastatic inducers. Importantly, pharmacological inhibition of YAP impairs mtp53 driven proliferation suggesting that YAP could be a central target for drug development to dismantle the oncogenic YAP/Hippo-p53 signaling (Di Agostino et al., 2016). Thus, further understanding of the complex pattern of interactions between YAP/Hippo and p53 family could be of great interest in dissecting cancer onset and ultimately, designing new anticancer strategies that could concomitantly target both pathways.

## Author Contributions

Both authors listed have made a substantial, direct and intellectual contribution to the work, and approved it for publication.

## Conflict of Interest Statement

The authors declare that the research was conducted in the absence of any commercial or financial relationships that could be construed as a potential conflict of interest.
